# Transposable Element Dysregulation in Fetal Down Syndrome Mesenchymal Stem Cells: Insights from a Model of Premature Ageing

**DOI:** 10.3390/ijms27219747

**Published:** 2026-10-30

**Authors:** Salvatore Vaiasicca, Laura Cianfruglia, Roberto Campagna, Monia Cecati, Maurizio Cardelli, Tiziana Casoli, Fabiola Olivieri, Stefania Fumarola

**Affiliations:** 1 Center of Biogerontology, Istituto Nazionale di Ricovero e Cura dell’Anziano, Istituto a Carattere Scientifico (IRCCS INRCA), Via Birarelli 8, 60121 Ancona, Italy; s.vaiasicca@inrca.it (S.V.); m.cardelli@inrca.it (M.C.); s.fumarola@inrca.it (S.F.); 2 Department for the Promotion of Human Science and Quality of Life, San Raffaele Roma University, Via di Val Cannuta, 247, 00166 Rome, Italymonia.cecati@uniroma5.it (M.C.); 3 Department of Clinical and Molecular Sciences, Università Politecnica delle Marche, 60126 Ancona, Italy

**Keywords:** Down syndrome, transposable elements, LINE-1, aging, mesenchymal stem cells

## Abstract

Down syndrome (DS) is associated with accelerated biological aging, but the molecular alterations linking trisomy 21 (T21) to early aging-related phenotypes remain incompletely understood. Transposable elements (TEs), particularly LINE-1 (L1) retrotransposons, are sensitive indicators of epigenetic and chromatin instability; however, their dysregulation during fetal development in DS remains largely unexplored. In this study, we performed a bioinformatic analysis of available RNA-sequencing data from chorionic villus-derived fetal mesenchymal stem cells (CV-MSCs) affected by T21 and compared them with data from euploid CV-MSCs. TE expression was quantified using TEtranscripts and analyzed across major TE classes, subfamilies, chromosomes, and evolutionary L1 groups, alongside curated gene-set analyses. At the class level, TE expression showed modest and heterogeneous differences between DS and CTRL samples. Among LINE-1 elements, ancient families, particularly HAL1 and L1M, showed a tendency toward higher expression, whereas L1PA2 and L1HS showed no evidence of increased expression. Pathway analysis revealed downregulation of pathways involved in heterochromatin maintenance and L1 repression, alongside alterations in DNA methylation, the DNA damage response, inflammatory/SASP pathways, nuclear architecture, and mesenchymal commitment. These findings highlight alterations in TE regulation and chromatin-related pathways in fetal DS cells, suggesting that molecular features potentially relevant to accelerated aging may be detectable early during development.

## 1. Introduction

Aging is increasingly recognized as a lifelong biological process rather than a phenomenon restricted to late adulthood. The progressive accumulation of molecular and cellular alterations arises from the complex interplay between genetic, epigenetic, and environmental factors throughout life; furthermore, early developmental stages are now considered critical for establishing the biological trajectories that influence health, disease susceptibility, and aging later in life [1,2]. During embryogenesis, environmental and epigenetic signals regulate chromatin organization and genome stability, highlighting the importance of fetal development in shaping long-term biological outcomes [3].

Maternal environmental influences represent one of the major determinants of fetal development. From the earliest stages of embryogenesis until birth, fetal development is shaped by a continuous exchange of molecular signals from the mother. This maternal–fetal communication orchestrates key developmental processes and can exert long-lasting effects on offspring health [4]. These molecular interactions occur primarily at the maternal–fetal interface, where chorionic villi (CV) play a fundamental role in nutrient exchange, immune regulation, and developmental signaling, particularly during the first trimester of pregnancy [5]. Consequently, cells isolated from CV provide a unique experimental model for investigating the earliest molecular events occurring during human development.

Down syndrome (DS), also known as trisomy 21 (T21), is the most common chromosomal aneuploidy in humans, affecting approximately 1 in 800 live births worldwide [6]. In approximately 95% of cases, DS results from maternal meiotic nondisjunction, leading to the presence of an extra copy of human chromosome 21 (HSA21). The resulting gene dosage imbalance profoundly alters developmental programs, giving rise to the characteristic clinical features of DS, including craniofacial dysmorphism, hypotonia, cognitive impairment, and multiple systemic manifestations [7].

Beyond its developmental phenotype, DS is widely recognized as a human model of accelerated aging. Individuals with DS develop several age-associated conditions significantly earlier than the general population, including premature skin wrinkling, hair graying, hearing loss, immune dysfunction, early menopause, and Alzheimer’s disease-like dementia [8]. However, aging in DS is not merely premature but also atypical and segmental, as only selected physiological systems undergo accelerated decline. Accordingly, DS has been classified as a progeroid syndrome characterized by the early onset of several disorders commonly associated with physiological aging [9,10,11].

Although the extra copy of chromosome 21 represents the primary genetic defect, DS induces transcriptomic alterations extending well beyond chromosome 21 itself. Indeed, the supernumerary chromosome affects not only HSA21 genes but also the global transcriptome through secondary effects of gene dosage imbalance and the presence of additional genetic material [12]. Since widespread transcriptomic remodeling represents one of the molecular signatures of biological aging, understanding the mechanisms responsible for these alterations may provide crucial insights into the accelerated aging phenotype observed in DS.

Repetitive elements (REs) constitute nearly half of the human genome and include transposable elements (TEs), which are capable of mobilizing throughout the genome [13]. Under physiological conditions, the vast majority of TEs are maintained in a transcriptionally silent state through epigenetic mechanisms, primarily DNA methylation and heterochromatin organization. However, the progressive loss of epigenetic control during aging results in the derepression of these repetitive elements, thereby contributing to genomic instability, chronic inflammation, and cellular senescence [14]. Although transcriptomic alterations associated with aging have been extensively investigated, the contribution of repetitive element dysregulation to accelerated aging remains poorly understood [15].

TEs can be broadly classified into retrotransposons, which mobilize through an RNA intermediate, and DNA transposons [16,17]. Among retrotransposons, long interspersed nuclear elements-1 (LINE-1 or L1) are of particular interest because, unlike most human TEs, a subset of evolutionarily young L1 elements retains autonomous retrotransposition capacity [18]. In contrast, many older TE families have accumulated disabling mutations and lost the ability to mobilize, although their sequences may have been co-opted by the host genome for regulatory functions [18,19].

Although historically considered genomic parasites, TEs are increasingly recognized as important components of genome regulation [20]. TE-derived sequences can contribute to transcriptional regulation, chromatin organization, and the generation of regulatory RNAs; therefore, their expression is tightly controlled by multiple layers of epigenetic repression, including DNA methylation, histone modifications, and heterochromatin formation [21,22]. Disruption of these mechanisms can result in TE transcriptional derepression and may affect genome stability and cellular homeostasis.

Increasing evidence also indicates that TE dysregulation is closely associated with cellular senescence and aging [23]. Age-related alterations in DNA methylation and chromatin organization can compromise the repression of endogenous retroelements, resulting in increased TE expression and potentially contributing to genomic instability and chronic inflammatory signaling [24,25,26]. Cardelli and colleagues highlighted the potential role of endogenous retroelements in cellular senescence and age-related diseases, placing these elements at the intersection of epigenetic instability, inflammation, and aging [23]. More broadly, recent studies have further supported the value of RE profiling for investigating molecular alterations associated with aging and age-related diseases, including through sequencing-based approaches for characterizing the human repeatome [13,27,28].

Importantly, TE transcriptional derepression should not necessarily be interpreted as evidence of active retrotransposition. Increased TE RNA levels may occur without subsequent genomic mobilization, particularly for evolutionarily ancient and retrotransposition-incompetent families [29,30]. Thus, the analysis of TE expression can provide crucial information about alterations in chromatin and epigenetic homeostasis, even in the absence of demonstrable insertion events. This distinction is particularly relevant when investigating early developmental tissues, in which alterations in epigenetic regulation may precede the accumulation of overt genomic damage.

DNA methylation is one of the major mechanisms responsible for preserving genome integrity through the regulation of RE activity. DNA methylation patterns are tightly regulated in a tissue-specific manner; global hypomethylation contributes to chromosomal instability and aberrant gene expression, whereas hypermethylation is generally associated with transcriptional silencing [31]. In particular, hypomethylation of subtelomeric and pericentromeric regions has been proposed to impair chromosome segregation during maternal meiosis, potentially contributing to chromosome 21 nondisjunction [32]. Furthermore, environmentally induced epigenetic modifications established during embryonic development may influence chromatin organization and genome stability [33].

Mesenchymal stem cells isolated from chorionic villi (CV-MSCs) represent an attractive model for investigating the earliest molecular alterations associated with T21. Since these cells originate from the fetal compartment during the first trimester of pregnancy, they retain the fetal genetic background while reflecting the developmental environment in which maternal molecular signals and epigenetic mechanisms actively shape embryonic development.

Despite the accumulating evidence describing transcriptomic and epigenetic alterations in DS, whether RE dysregulation is already established during fetal development remains largely unknown. This question is particularly relevant in the context of the accelerated aging phenotype associated with DS, as alterations in chromatin organization, DNA methylation, genome stability, and cellular senescence are also recognized features of biological aging. The early identification of molecular alterations affecting TE regulation could therefore provide insights into mechanisms that are established during development and may subsequently contribute to age-related cellular dysfunction.

In the present study, we investigated TE transcriptional profiles in DS-MSCs and compared them with those of their euploid counterparts (CTRLs), using a secondary bioinformatic analysis of publicly available RNA-sequencing data. We characterized TE expression at the level of major TE classes, subfamilies, chromosomes, and evolutionary L1 groups, with particular attention to the differential behavior of ancient and evolutionarily young L1 families. We further investigated the expression of gene networks involved in heterochromatin maintenance, DNA methylation, L1 repression, nuclear architecture, the DNA damage response, innate immune signaling, cellular senescence, inflammatory and senescence-associated secretory phenotype (SASP) pathways, and mesenchymal lineage commitment. Finally, we explored chromosome-matched relationships between pathway-associated genes and L1 expression. Through this integrated analysis, we aimed to determine whether alterations in TE regulation and the molecular systems responsible for maintaining epigenetic and genomic integrity are already detectable during fetal development in DS, and to assess their potential relevance to the molecular mechanisms underlying accelerated aging.

## 2. Results

### 2.1. Transposable Element Classes Show Distinct Expression Patterns in DS-MSCs

TE expression was first examined at the level of major TE classes in DS and CTRL CV-MSCs. Normalized TE expression values were aggregated at the sample level for each TE class, with the three DS and three CTRL samples treated as biological replicates (Figure 1). At the TE-class level, LINE and satellite elements exhibited higher mean expression in DS samples compared with CTRLs, with differences of 0.10 (5.37 vs. 5.26) and 0.35 (3.66 vs. 3.31) log2-CPM, respectively. In contrast, DNA and LTR elements showed minimal differences between groups (3.75 vs. 3.73 and 3.50 vs. 3.46, respectively), while SINE and OTHER elements displayed slightly lower expression in DS samples (6.39 vs. 6.46 and 4.09 vs. 4.20, respectively). However, none of these differences reached statistical significance after correction for multiple testing. Overall, these findings suggest modest, class-dependent variations in TE expression between DS and CTRL samples, rather than a consistent alteration across all TE classes (Figure 1).

To further characterize the class-level expression patterns, we examined TE expression at the subfamily level within each major TE class (Figure 2). Among SINEs, Alu elements showed a slight negative effect, whereas MIR and OTHER SINEs showed values close to zero, with broad confidence intervals. Within DNA transposons, most subfamilies showed small positive or near-neutral effects, although the confidence intervals indicated substantial variability between samples. For LINEs, L1 showed a positive effect, whereas L2 and the aggregated OTHER category showed more variable patterns. Among LTR elements, ERVL-MaLR, ERVL, ERV1, and ERVK showed modest positive effects, while OTHER and Gypsy displayed smaller or negative effects. Finally, satellite subfamilies showed heterogeneous patterns, with Telomeric and Satellites. Other showing positive effects, whereas Centromeric and Acrocentric elements were closer to zero. Overall, none of the subfamily-level comparisons reached statistical significance after correction for multiple testing, indicating that the observed differences between individual TE subfamilies should be considered descriptive and exploratory.

### 2.2. TE Expression in DS Exhibits Chromosome-Specific Patterns

To determine whether TE expression patterns were uniformly distributed across the genome, TE expression was further examined according to chromosome and TE class (Figure 3).

For each chromosome–TE class combination, normalized expression values were aggregated at the sample level to calculate the mean log2FC between DS and CTRL samples.

This chromosome-resolved analysis revealed substantial heterogeneity across genomic regions. LINE elements showed generally positive log2FC values across most chromosomes, with a more pronounced increase on chromosome 21. Similarly, SINE, DNA, and LTR elements showed modest and variable changes across chromosomes, with relatively higher values on chromosome 21. In contrast, satellite elements displayed the greatest variability, with marked positive or negative effects on specific chromosomes. However, these findings should be interpreted cautiously, as some chromosome–class combinations included only a limited number of TE entries. The OTHER category showed smaller and less consistent changes across chromosomes. Overall, these results suggest that TE expression changes in DS are not uniformly distributed across the genome but differ according to both TE class and chromosomal location.

### 2.3. Evolutionarily Distinct L1 Families Display Different Expression Patterns

To gain further insight into the LINE alterations, L1 elements were subsequently examined according to their evolutionary age. L1 loci were classified into five evolutionary groups: HAL1, L1M, L1P-other, L1PA2, and L1HS. Importantly, because the TEtranscripts output aggregates TE expression across annotated loci within chromosomes, the number of entries associated with each evolutionary group reflects the structure of the TE annotation and chromosome-level aggregation rather than the number of individual genomic insertions. Thus, L1HS and L1PA2 correspond to specific L1 subfamilies, whereas HAL1, L1M and L1P-other represent broader evolutionary groups in which multiple L1 subfamilies are aggregated. Consequently, the different numbers of entries assigned to each group (HAL1, *n* = 86; L1M, *n* = 1662; L1P-other, *n* = 610; L1P2, *n* = 24; L1HS, *n* = 24) reflect the different levels of evolutionary grouping and should not be interpreted as equivalent numbers of individual L1 insertions. For the statistical analysis, normalized expression values were aggregated within each evolutionary group at the sample level, with the three Down syndrome and three control samples treated as biological replicates.

HAL1 and L1M showed positive mean log2FC values (0.295 and 0.170, respectively), whereas L1P-other showed a near-neutral effect (−0.056). In contrast, L1PA2 showed a marked negative mean log2FC (−0.534), while L1HS also showed a negative effect (−0.499). However, none of these differences reached statistical significance after correction for multiple testing. Overall, these results suggest a tendency toward increased expression of older L1 evolutionary groups and reduced expression of younger L1 groups, although this pattern should be considered exploratory given the limited number of biological replicates (Figure 4).

### 2.4. Pathway Analysis Identifies Disruption of Chromatin and TE-Repression Programs with Coordinated Transcriptional Alterations

We next investigated whether these TE alterations occurred in parallel with changes in cellular pathways related to chromatin regulation, genome stability, and cellular aging. Gene-set analysis was performed using CAMERA, a competitive gene-set test that accounts for inter-gene correlation. The enrichment analysis showed strong enrichment of downregulated genes within gene sets involved in heterochromatin organization and L1 repression, followed by significant enrichment of downregulated genes within pathways involved in DNA damage response, inflammatory SASP, DNA methylation, and mesenchymal commitment. In contrast, the senescence pathway showed a positive enrichment trend, although this signal was substantially weaker than the downregulation observed for chromatin-related pathways. The size of each point represents the percentage of significant genes within the corresponding pathway, while the x-axis represents the signed −log10(FDR) (Figure 5A).

To visualize the genes contributing to the major pathway-level changes, we generated hierarchical clustering heatmaps using normalized gene expression values. The DNA methylation maintenance gene set included seven genes, with several DNA methyltransferases and *TET*-family genes showing reduced expression in DS samples relative to controls (Figure 5B). The L1 repression gene set comprised 15 genes, including chromatin modifiers and repressors such as *EZH1*, *EZH2*, *TRIM28*, *MORC2*, *KDM1A*, *KDM4A*, and *SETDB1*, and showed an overall shift toward lower expression in DS-MSCs (Figure 5C). A similar pattern was observed for the DNA damage response (DDR) pathway (13 genes), in which most genes—including *BRCA1*, *BRCA2*, *RAD51*, *MDC1*, *CHEK1*, *ATR*, *PALB2*, and *MRE11*—displayed lower expression in DS samples (Figure 5D). The inflammatory SASP gene set (12 genes) also showed substantial transcriptional differences, involving genes such as *IL1A*, *IL6*, *CXCL8*, *NFKB1*, *RELA*, *JUN*, and *MAPK1/3/7* (Figure 5E). The mesenchymal lineage commitment pathway (15 genes) displayed coordinated changes involving transcriptional regulators, including *TWIST2*, *PRRX1*, *RUNX2*, *SOX*-family genes, and *PPARG* (Figure 5F). Finally, the heterochromatin maintenance pathway gene set showed a strong enrichment of downregulated genes, including *CBX1*, *CBX3*, *CBX5*, *SUV39H1*, *SUV39H2*, *LMNB1*, *EHMT1*, and *HELLS* (Figure 5G).

### 2.5. Pathway-Associated Genes Show a Non-Uniform Genomic Distribution

To investigate the chromosomal distribution of these transcriptional alterations, pathway-associated genes were mapped to their genomic positions (Figure 6).

The genomic map showed that pathway-associated genes were distributed across multiple chromosomes, with several chromosomes containing genes belonging to more than one pathway. The analysis included genes involved in heterochromatin maintenance, L1 repression, DNA methylation, the DNA damage response, the inflammatory SASP, cGAS–STING, senescence, and mesenchymal commitment.

Chromosomal enrichment was evaluated using 2 × 2 Fisher’s exact tests, comparing the proportion of significant versus non-significant pathway genes located on each chromosome with the corresponding distribution across all other chromosomes. Expected numbers were calculated from the genomic distribution of the pathway-gene background. The resulting observed/expected ratios revealed chromosome-specific enrichment and depletion patterns, indicating that the significant pathway-associated genes were not uniformly distributed throughout the genome (Figure S1).

## 3. Discussion

DS is characterized by extensive transcriptional and epigenetic alterations that affect more than just the direct gene-dosage effects of chromosome 21 [34,35]. Although these changes have been extensively investigated in postnatal and adult tissues, the regulation of RE, particularly during early fetal development, remains comparatively less explored. In the present study, we investigated the expression of TEs together with the transcriptional pathways involved in chromatin regulation, genome stability, and cellular aging in CV-MSCs of fetuses with T21. Our findings reveal a modest and non-uniform alteration in TE expression in fetal CV-MSCs from fetuses with T21. At the class level, TE expression showed heterogeneous differences between DS and CTRL samples, but no major TE class reached statistical significance after multiple-testing correction. At the same time, pathway analysis identified coordinated alterations in genes involved in heterochromatin maintenance, L1 repression, DNA methylation, and the DNA damage response. Together, these findings suggest that alterations in chromatin-regulatory pathways are detectable during fetal development in DS, while the TE transcriptional changes themselves appear more selective and modest.

One of the major findings of the present study is the coordinated downregulation of genes involved in heterochromatin maintenance and TE repression. These results support the growing body of evidence suggesting that epigenetic remodeling is a fundamental component of DS pathogenesis, rather than simply a secondary consequence of aging [35,36,37]. Transcriptomic and epigenetic studies have shown that T21 affects gene regulatory networks from the early stages of fetal development [38], leading to widespread alterations in chromatin organization [39], DNA methylation [40,41], and transcriptional regulation [42,43]. These molecular alterations have also been proposed to contribute to the accelerated biological aging phenotype associated with DS. In this context, RE dysregulation has recently emerged as a potential molecular component linking T21, epigenetic instability, and age-related phenotypes [44]. The reduction of heterochromatin-related pathways observed in our dataset is particularly relevant considering the central role of constitutive heterochromatin in maintaining genomic stability through the silencing of repetitive DNA [45]. The loss of heterochromatin integrity is a well-recognized hallmark of cellular senescence and physiological aging, and it has been associated with the transcriptional derepression of TEs [46]. However, these pathway-level alterations should not be interpreted as evidence of a direct causal relationship between chromatin deregulation and TE expression. Rather, they identify a transcriptional context in which TE regulation may be altered.

Importantly, previous studies provide both broad and gene-specific evidence of altered gene regulation in DS. Letourneau et al. reported widespread domains of transcriptional dysregulation associated with changes in the chromatin environment in fetal fibroblasts, supporting the notion that trisomy 21 can affect gene regulation beyond chromosome 21 [34]. At the gene level, Shi et al. reported reduced *EZH2* expression in fetal hippocampal tissue from individuals with DS, providing a relevant precedent for the reduced expression of this chromatin regulator observed in our L1 repression gene set [47]. Alterations in DNA methylation-related mechanisms have also been described in peripheral blood mononuclear cells from individuals with DS. Ciccarone et al. reported reduced expression of *DNMT3A*, *TET1,* and *TET3*, whereas *DNMT1* and *DNMT3B* expression did not differ significantly between DS and controls [48]. Together, these studies support the involvement of chromatin regulation and DNA methylation in DS, while also highlighting that changes are gene- and tissue-dependent. Our findings extend this evidence by identifying altered expression patterns in chromatin maintenance, L1 repression, and DNA methylation-related pathways in fetal CV-MSCs. However, given the limited sample size and the absence of independent validation, these results should be interpreted as pathway-level transcriptional alterations rather than evidence of uniform dysregulation of all pathway components.

In our dataset, these alterations were not restricted to heterochromatin maintenance but also involved pathways related to DNA methylation, L1 repression, and the DNA damage response. This broader pattern is consistent with alterations in multiple interconnected mechanisms involved in the maintenance of epigenetic and genomic stability. Importantly, these findings do not establish whether altered TE expression is a consequence of chromatin dysregulation, contributes to it, or reflects a common upstream regulatory change. Notably, these alterations are likely not driven solely by the direct gene-dosage effect of the extra chromosome 21. Increasing evidence indicates that T21 induces genome-wide transcriptional and epigenetic changes, affecting regulatory networks that involve genes located both on HSA21 and throughout the rest of the genome [49,50,51].

Consistent with the genome-wide transcriptional dysregulation previously described in T21 [34,49], our chromosome-resolved analysis showed that TE deregulation was distributed across multiple chromosomes rather than being confined to chromosome 21, although the composition of the response varied among genomic regions. LINE elements showed a generally positive expression trend across chromosomes, with a more pronounced pattern on chromosome 21, whereas SINE and LTR elements displayed more modest and variable differences. Satellite elements showed the greatest heterogeneity, which should be interpreted cautiously because several chromosome–class combinations were based on a limited number of elements. These descriptive findings suggest that the transcriptional consequences of T21 may extend to RE distributed throughout the genome, although the chromosome-level analysis was exploratory and was not subjected to independent statistical testing. These findings extend the concept of genome-wide transcriptional dysregulation in DS to RE and are consistent with emerging evidence indicating altered TE expression, particularly of L1 in DS [36].

A particularly interesting feature emerged from the evolutionary stratification of L1 elements. Rather than showing uniform activation across all L1 families, the sample-level analysis indicated a tendency toward higher expression of the evolutionarily older L1 groups, particularly HAL1 and L1M, whereas the younger L1PA2 and L1HS groups did not show significant increases after multiple-testing correction. This pattern should be interpreted cautiously, as none of the five evolutionary L1 groups reached statistical significance after correction for multiple comparisons. Nevertheless, the direction of the observed changes suggests that TE expression in fetal DS-MSCs may be evolutionarily selective rather than reflecting a generalized activation of L1 elements.

This distinction is biologically relevant since L1HS and recent L1PA2 elements represent the youngest L1 families, whereas HAL1 and L1M represent substantially older evolutionary groups that have accumulated mutations and are generally no longer capable of autonomous retrotransposition. Therefore, the tendency toward higher expression of HAL1 and L1M should not be interpreted as evidence of increased retrotransposition. Instead, their altered expression may reflect increased transcriptional accessibility of genomic regions containing these ancient repetitive sequences in the context of disrupted chromatin regulation. Importantly, the detected transcripts may not necessarily correspond to autonomous L1-derived transcripts, but may instead represent L1-derived sequences incorporated into the transcripts of host genes or non-coding RNAs. It is also important to note the absence of significant global L1HS activation. L1 regulation depends on the cellular context; Macia et al. demonstrated that human MSCs exhibit markedly lower L1 expression and retrotransposition activity than neural progenitor cells, suggesting efficient restriction of retrotransposition-competent L1 elements in the mesenchymal lineage [52]. Therefore, the absence of substantial L1HS deregulation in our dataset may be compatible with the intrinsic regulation of L1 in MSCs, suggesting that chromatin remodeling alone may not be sufficient to overcome the multiple safeguards controlling young L1 families. However, it cannot be concluded that young L1 elements remain completely unaffected in T21, particularly because family-level RNA-seq analyses cannot resolve the behavior of individual retrotransposition-competent loci.

An additional biological confounder should be considered when interpreting the chromatin- and DNA damage-related findings. Several of the downregulated genes identified in these pathways, including *BRCA1*, *RAD51*, *EZH2*, *DNMT1,* and *LMNB1*, are also associated with cell proliferation and cellular growth state. Importantly, the same DS and euploid CV-MSC samples analyzed in the present study were previously characterized as exhibiting reduced proliferative capacity and altered cell-cycle distribution [50]. Thus, differences in cellular growth state between DS and CTRL cultures may contribute, at least in part, to the transcriptional pattern observed here. Although the present dataset does not include direct measures of proliferation or cellular senescence, the lack of significant enrichment of the curated senescence gene set argues against interpreting these findings as evidence of an established senescent phenotype.

Importantly, the fetal origin of the MSCs analyzed in the present study provides a crucial context for interpreting these findings. Most studies investigating transcriptional and epigenetic alterations in DS have focused on postnatal tissues or adult individuals, where molecular changes may reflect a combination of primary trisomy-associated alterations, aging, and disease progression [42]. In contrast, CV-MSCs represent an early developmental stage preceding many of the neurological, immunological, and degenerative manifestations associated with DS. The detection of altered heterochromatin maintenance, DNA methylation, TE repression, and RE expression in these cells therefore indicates that alterations in chromatin regulation are already detectable during fetal development. These observations are consistent with the possibility that some molecular features associated with the later aging phenotype of DS may be established early in development, although the present study cannot establish a causal link between these fetal alterations and accelerated aging.

Several limitations should be considered when interpreting these findings. First, the relatively limited number of fetal CV-MSC samples restricts statistical power, particularly for exploratory class-, subfamily-, and evolutionary-group analyses. Second, the original RNA-sequencing libraries were generated using poly(A) selection, which preferentially captures polyadenylated transcripts and may underrepresent non-polyadenylated or inefficiently polyadenylated TE-derived RNAs. The TE profiles described here therefore represent the detectable polyadenylated fraction of the TE transcriptome rather than its complete transcriptional landscape. Third, TE expression was quantified using TEtranscripts and subsequently summarized at the family and chromosome levels. Although this approach enables the analysis of repetitive element expression from conventional RNA-seq data, it does not resolve individual genomic insertions and therefore cannot determine whether specific retrotransposition-competent L1 loci are differentially regulated. These methodological considerations are particularly relevant when interpreting the absence of global L1HS or L1PA2 activation. A further limitation is that differences in developmental state cannot be completely excluded. Although all samples were obtained within the same general gestational window, the present dataset does not include independent molecular markers that would allow a precise assessment of developmental equivalence between DS and CTRL CV-MSCs. Thus, some of the modest transcriptional differences observed may potentially reflect variation in cellular developmental state. Finally, the relatively modest class-level differences in TE expression should be interpreted primarily as molecular signatures rather than evidence of functionally relevant TE activation. Whether these changes have biological consequences remains to be established experimentally. In addition, the present analysis did not distinguish between sense and antisense transcription of individual TE loci. Because TEtranscripts aggregates reads according to RepeatMasker annotations, strand-specific interpretation of TE-derived transcripts was beyond the scope of the present analysis.

Future studies using a larger fetal cohort, alongside TE-focused and locus-specific sequencing approaches, will therefore be crucial to validate and extend these observations. These investigations should ideally examine both evolutionarily ancient LINE families—such as HAL1 and L1M, which emerged as the predominant deregulated families in the present study—and younger, potentially retrotransposition-competent elements, particularly L1HS and L1PA2. In particular, locus-specific characterization of L1HS elements may reveal alterations that are obscured by family- or chromosome-level aggregation, helping to determine whether individual active L1 loci escape epigenetic repression during early fetal development. Furthermore, the integration of TE-specific transcriptomics with chromatin accessibility, DNA methylation, and histone modification profiling would help establish whether the transcriptional alterations identified here directly reflect changes in the epigenetic landscape. Functional studies of MSC differentiation and cellular senescence could further determine the biological consequences of the pathway alterations observed in fetal DS cells.

Given the limited availability and biological value of fetal CV-MSCs, the present study should be considered a first exploratory characterization of TE transcriptional deregulation in DS during intrauterine development. Rather than providing a comprehensive assessment of TE activity, our findings identify an early molecular pattern characterized by the preferential deregulation of evolutionarily ancient LINE families in the context of altered chromatin and epigenetic regulatory pathways. This provides a clear rationale for future TE-focused and locus-specific investigations, highlighting the potential value of fetal DS samples for understanding how early epigenetic instability may contribute to the subsequent emergence of accelerated aging phenotypes. Overall, our findings identify an early molecular pattern in fetal DS characterized by altered chromatin-regulatory pathways together with modest and evolutionarily selective differences in TE expression. The preferential tendency toward higher expression of older L1 families, particularly HAL1 and L1M, in the absence of global L1HS or L1PA2 activation suggests that TE dysregulation in fetal DS MSCs is not uniform across evolutionary families. Instead, it occurs within a broader context of altered heterochromatin maintenance, DNA methylation, genome surveillance, and developmental transcriptional programs. These observations provide a basis for investigating whether early alterations in chromatin and RE regulation contribute to the molecular trajectory linking T21 with later aging-related phenotypes.

## 4. Materials and Methods

### 4.1. Study Design and RNA-Seq Dataset

This study represents a secondary bioinformatic analysis of publicly available RNA-sequencing data generated by Vaiasicca et al. [50]. No biological samples were collected or processed as part of the present study. Raw RNA-seq data were retrieved from the Gene Expression Omnibus (GEO) repository (accession number GSE184450) and reanalyzed to investigate the expression profiles of TEs. The original dataset comprised mesenchymal stem cells isolated from chorionic villi (CV-MSCs) obtained during routine prenatal diagnosis from women carrying fetuses with T21 (DS, *n* = 3) and euploid fetuses (CTRLs, *n* = 3). Chorionic villi were collected between the 10th and 13th weeks of gestation. Complete details regarding sample preparation, sequencing procedures, and quality control are available in the original publication [50].

### 4.2. Transposable Element Quantification

Raw RNA-sequencing data (FASTQ files) were downloaded and reanalyzed using the Galaxy Europe platform (https://usegalaxy.eu, accessed on 23 January 2026). Sequence reads from each sample were independently aligned to the human reference genome GRCh38 (hg38) using the RNA STAR tool and the pre-indexed hg38 reference genome. Single-end reads (75 bp) were aligned using the STAR setting specified in the Galaxy workflow. The maximum number of genomic alignments considered per read was set to 100 (outFilterMultimapNmax = 100), and all reported multimapping alignments were retained in the BAM output (outSAMmultNmax = −1).

TEtranscripts (version 2.2.3+galaxy0) was used to generate gene- and TE-level count matrices, using the Illumina TruSeq stranded (first-strand/reverse stranded) library setting and the multi-mapping mode. The minimum read count cutoff was set to 10, and multimapping read assignment was optimized over 100 iterations. The fragment-length parameter was left unspecified, consistent with the default behavior for single-end data.

Gene annotations were obtained from GENCODE for hg38/GRCh38, whereas transposable element (TE) annotations were obtained from the RepeatMasker (rmsk) table for hg38 downloaded from the UCSC Genome Browser. Subsequent normalization and differential expression analyses were performed in R using the limma package, as described below.

### 4.3. Differential Expression Analysis of Transposable Elements

TEtranscripts generated a count matrix (cntTable) reporting the raw read counts assigned to each annotated gene and TE. TEs were first filtered by retaining only those elements with at least one read in at least one DS sample and at least one CTRL sample. To account for differences in sequencing depth and RNA composition among samples, raw TE counts were normalized using the trimmed mean of M-values (TMM) method implemented in the edgeR package. Normalized expression values were subsequently transformed using the voom method implemented in the limma package, generating log_2_-counts per million (log_2_-CPM) values with precision weights.

To investigate TE deregulation at different hierarchical levels, individual elements were grouped according to their RepeatMasker annotations into the major TE classes (LINE, SINE, LTR, DNA transposons, Satellite, and Other) and subsequently classified into their respective subfamilies. For each TE class or subfamily, expression values were first aggregated at the sample level by calculating the mean normalized expression of all TEs belonging to the corresponding class or subfamily within each individual sample. Differences between DS and CTRL samples were then evaluated using two-sided Welch’s t-tests, with the three biological replicates in each group considered as independent samples. Resulting *p*-values were adjusted for multiple testing using the Benjamini–Hochberg FDR method, with multiple-testing correction performed separately within each TE class for the subfamily-level analyses.

For each TE class and subfamily, the log_2_FC was calculated as the difference between the mean log_2_-CPM across DS samples and the mean log_2_-CPM across CTRL samples, corresponding to the log_2_ fold change (DS/CTRLs). Ninety-five percent confidence intervals (95% CIs) were calculated from the sample-level values. Differential expression patterns of TE classes and subfamilies were visualized using customized bar plots showing the mean log_2_FC and 95% CIs, generated with the ggplot2 package in R (version 4.4.1).

### 4.4. Chromosome-Specific Transposable Element Analysis

To investigate the chromosomal distribution of TE deregulation, each TE was assigned to its genomic chromosome based on the RepeatMasker annotation generated by TEtranscripts. For each chromosome–TE class combination, normalized TE expression values were aggregated at the sample level by calculating the mean log_2_-CPM of all TEs belonging to the corresponding chromosome and TE class within each sample. The mean expression across DS and CTRL samples was then used to calculate the log_2_FC (DS/CTRLs). The resulting chromosome-by-class matrix was visualized as a heatmap using the ggplot2 package in R, enabling the identification of chromosome-specific patterns of TE deregulation. This analysis was considered descriptive and exploratory, and no independent statistical test was performed for individual chromosome–TE class combinations.

### 4.5. Evolutionary Analysis of L1 Subfamilies

To investigate whether LINE deregulation differed according to evolutionary age, elements annotated as LINE/L1 were further classified into the major RepeatMasker subfamilies according to their L1 family annotation extracted from the TE identifier. Five evolutionary groups were considered: HAL1, L1M, L1P-other, L1PA2, and L1HS. The L1P group was therefore subdivided into the specific L1PA2 subfamily and all other L1P elements (L1P-other).

For each evolutionary group, normalized TE expression values were aggregated at the sample level by calculating the mean log_2_-CPM of all L1 elements belonging to that group within each individual sample. Differential expression was then calculated as the log_2_FC between the mean expression across DS and CTRL samples.

For each L1 evolutionary group, differences between DS and CTRL samples were evaluated using two-sided Welch’s t-tests, with the three biological replicates in each group considered as independent samples. The resulting *p*-values were adjusted for multiple testing using the Benjamini–Hochberg method. Confidence intervals (95% CIs) were calculated for the estimated log_2_FC values. The number of annotated L1 elements belonging to each evolutionary group was also reported.

### 4.6. Pathway Enrichment Analysis

To investigate the biological processes associated with TE deregulation, a panel of custom gene sets was generated by integrating pathways available in the Molecular Signatures Database (MSigDB v2025.1) with manually curated genes derived from the literature. Specifically, the SASP pathway was based on the WikiPathways gene set WP_SENESCENCEASSOCIATED_SECRETORY_PHENOTYPE_SASP; DNA methylation maintenance and heterochromatin organization were assembled from the corresponding biological processes represented in the MSigDB collections (BioCarta, Canonical Pathways, and Gene Ontology) and refined to include genes with well-established roles in the maintenance of chromatin organization and epigenetic regulation. The senescence gene set was manually curated based on the Reactome Cellular Senescence pathway (R-HSA-2559583), focusing on genes involved in cell-cycle arrest and senescence-associated growth inhibition, including *CDKN1A*, *CDKN1B*, *CDKN2A*, *CDKN2B*, *CDKN2C*, *CDKN2D*, and *IGFBP7*. The gene set describing L1 epigenetic repression was manually curated according to the epigenetic regulators of L1 expression comprehensively reviewed by Liu et al., including factors involved in DNA methylation, histone modifications, chromatin remodeling, and RNA-mediated regulation of L1 silencing [53].

The DDR gene set was manually curated based on the major components of DNA damage sensing, checkpoint signaling, and DNA repair pathways described by Tiwari and Wilson [54]. The resulting gene set included *ATM*, *ATR*, *H2AFX*, *MDC1*, *MRE11*, *RAD50*, *NBN*, *CHEK1*, *CHEK2*, *TP53*, *BRCA1*, *BRCA2*, *PALB2*, and *RAD51*. The cGAS–STING gene set was manually curated based on the canonical cGAS–STING signaling pathway and its major upstream and downstream regulators, as described by Hopfner and Hornung [55], including *MB21D1*, *TMEM173*, *TBK1*, *IRF3*, *IRF7*, *TREX1*, and *IFI16*. The interferon/viral mimicry gene set was manually curated to capture the major components of cytosolic RNA sensing and interferon-stimulated responses, including *DDX58*, *IFIH1*, *MAVS*, *ISG15*, *MX1*, *OAS1*, *OAS2*, and *OAS3*. A curated mesenchymal lineage commitment gene set was generated by integrating the master transcriptional regulators consistently identified in three independent reviews of MSC differentiation, including Almalki and Agrawal [56] and Rauch and Mandrup [57], as well as the review on MSC dysfunction in DS [58]. Only transcription factors repeatedly recognized as central regulators of osteogenic, chondrogenic, adipogenic, or early mesenchymal lineage commitment were retained for downstream pathway analysis.

Gene set enrichment analysis was performed using the CAMERA (Correlation-Adjusted MEan RAnk) algorithm implemented in the limma package in R. CAMERA is a competitive gene set test that accounts for inter-gene correlation while using the same linear modeling framework adopted for differential gene expression analysis. Pathways with a False Discovery Rate (FDR) < 0.05 were considered significantly enriched.

To visualize pathway-specific transcriptional changes, genes belonging to selected pathways were extracted from the normalized expression matrix and displayed as heatmaps using the pheatmap package in R. Prior to visualization, expression values were standardized on a gene-by-gene basis by calculating row-wise \(z\)-scores, thereby emphasizing relative expression differences across biological samples independently of absolute expression levels. Hierarchical clustering was performed on genes, whereas samples were displayed according to their biological group.

## 5. Conclusions

In conclusion, our findings identify transcriptional alterations involving chromatin homeostasis and TE regulation detectable in DS-MSCs. The observed expression patterns of evolutionarily ancient L1 families, particularly HAL1 and L1M, were accompanied by alterations in heterochromatin organization, DNA methylation, L1 repression, DDR, inflammatory pathways, and mesenchymal lineage commitment. These findings suggest that transcriptional alterations involving chromatin and repeat element regulation are already detectable in fetal DS and may provide insights into molecular features potentially relevant to the aging-related phenotypes that emerge later in life. Further studies using larger fetal cohorts and TE-focused, locus-specific approaches will be required to validate these findings and determine the functional consequences of individual L1 alterations.

## Figures and Tables

**Figure 1:**
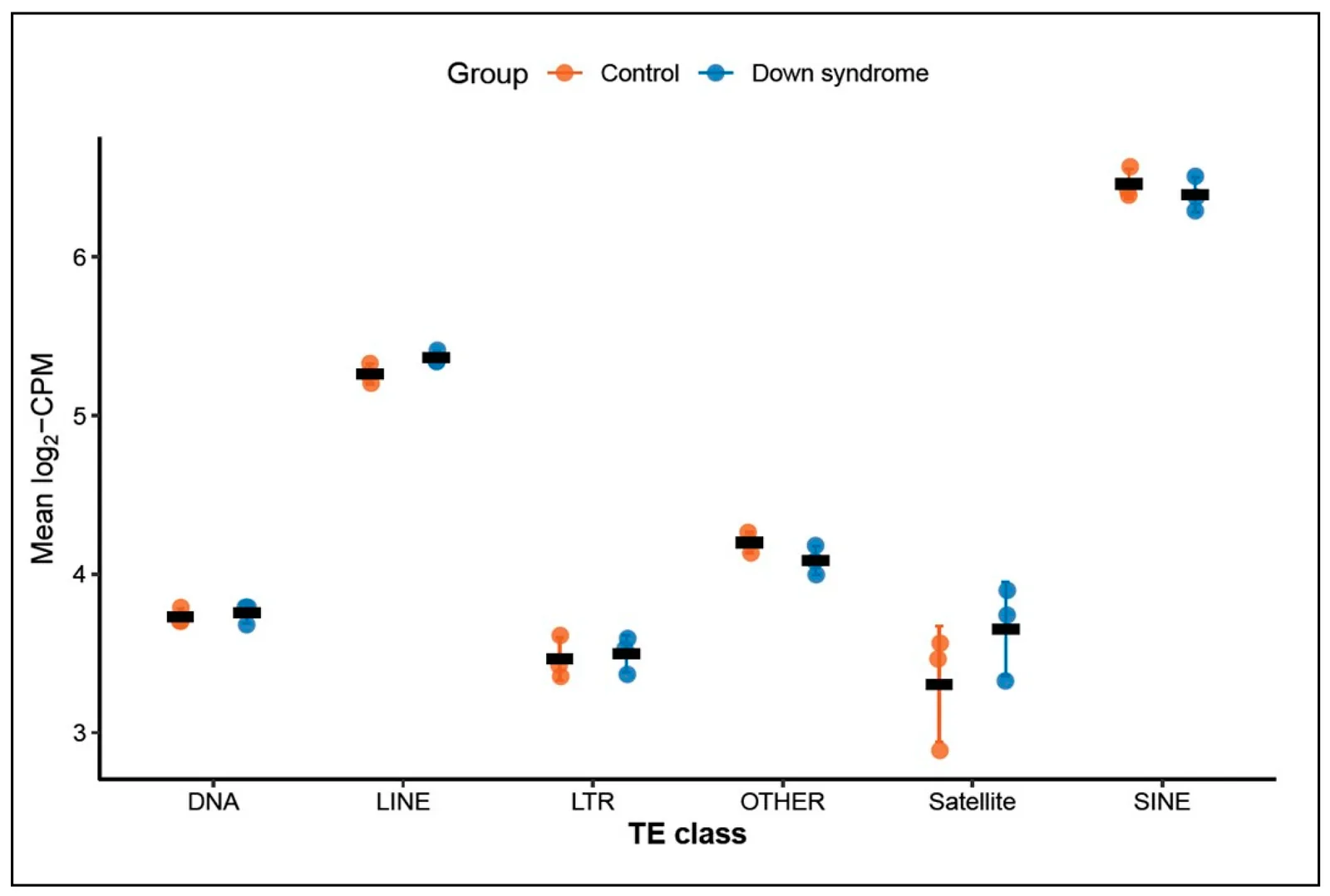
Expression patterns of major transposable element classes in DS-MSCs. The plot shows the mean normalized TE expression, expressed as log_2_-CPM, for the six major TE classes (DNA, LINE, LTR, OTHER, Satellite, and SINE). Each colored dot represents an individual biological sample (*n* = 3 per group). The black horizontal bars indicate the mean expression for each group, while the vertical error bars represent ±1 standard deviation (SD). Control samples are shown in orange, and DS samples in blue. The plot is descriptive and illustrates the distribution and variability of TE expression across biological replicates within each class.

**Figure 2:**
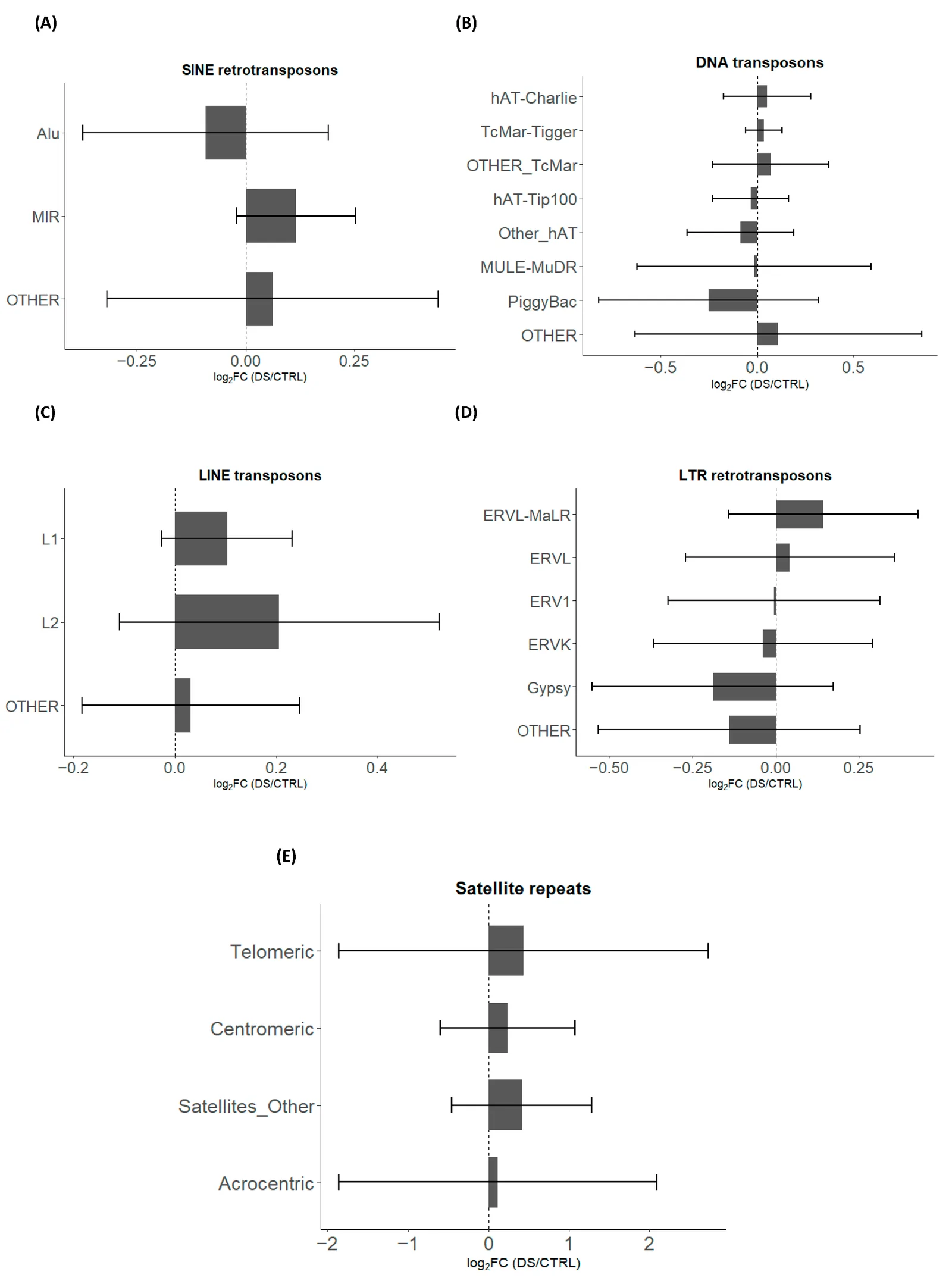
Expression patterns of TE subfamilies in DS-MSCs. Bar plots showing the mean log2 fold change (log2FC) in expression of TE subfamilies in DS compared with CTRL samples for SINEs (**A**), DNA transposons (**B**), LINEs (**C**), LTR retrotransposons (**D**), and satellite repeats (**E**). Positive log2FC values indicate higher expression in DS relative to CTRLs, whereas negative values indicate lower expression. Bars represent mean log2FC values, and error bars indicate 95% confidence intervals. Comparisons were performed using normalized expression values aggregated at the sample level, with three DS and three CTRL samples treated as biological replicates. Statistical significance was assessed after multiple-testing correction within each TE class; no subfamily-level comparison reached statistical significance.

**Figure 3:**
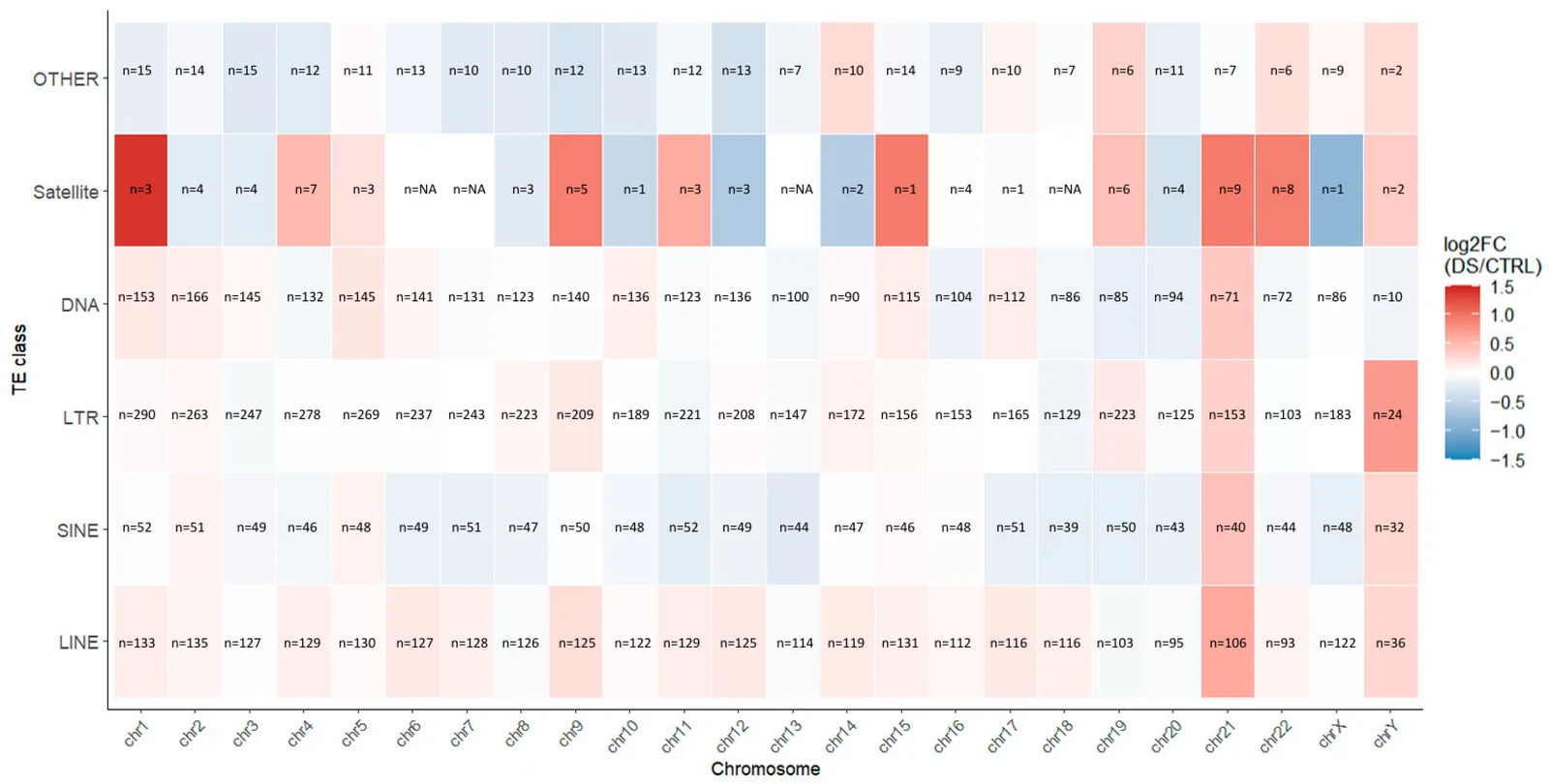
Chromosome-specific distribution of TE expression. Heatmap showing the mean log2 fold change (log2FC) in normalized TE expression between DS and CTRL samples for each chromosome–TE class combination. The number of TE entries contributing to each chromosome–TE class combination is indicated by n within each cell. NA indicates that no TE entries were annotated for the corresponding chromosome–class combination. TE expression values were aggregated at the sample level within each chromosome and TE class, and positive values indicate higher expression in DS relative to CTRLs, whereas negative values indicate lower expression. The analysis includes chromosomes 1–22, X, and Y and the major TE classes: LINE, SINE, LTR, DNA, Satellite, and OTHER.

**Figure 4:**
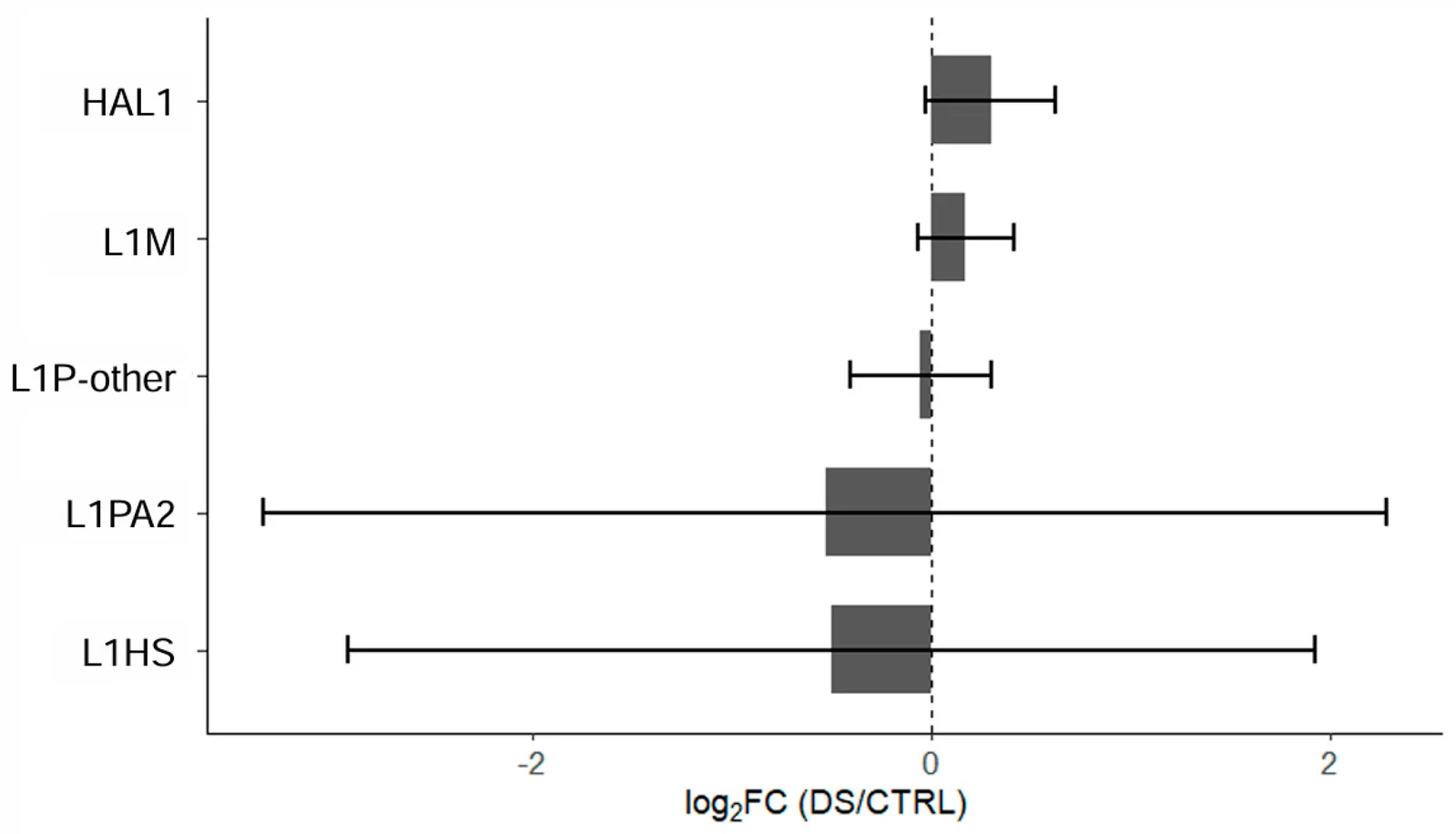
Evolutionary age-dependent patterns of L1 expression in DS cells. Bar plot showing the mean log2FC of L1 evolutionary groups in MSCs from individuals with DS compared with CTRLs. L1 elements were classified into five evolutionary groups: HAL1, L1M, L1P-other, L1PA2, and L1HS. The number of annotated L1 entries included in each evolutionary group is indicated in parentheses. Positive log2FC values indicate higher expression in DS relative to CTRLs, whereas negative values indicate lower expression. Bars represent mean log2FC values, and error bars indicate 95% confidence intervals. None of the comparisons reached statistical significance after correction for multiple testing.

**Figure 5:**
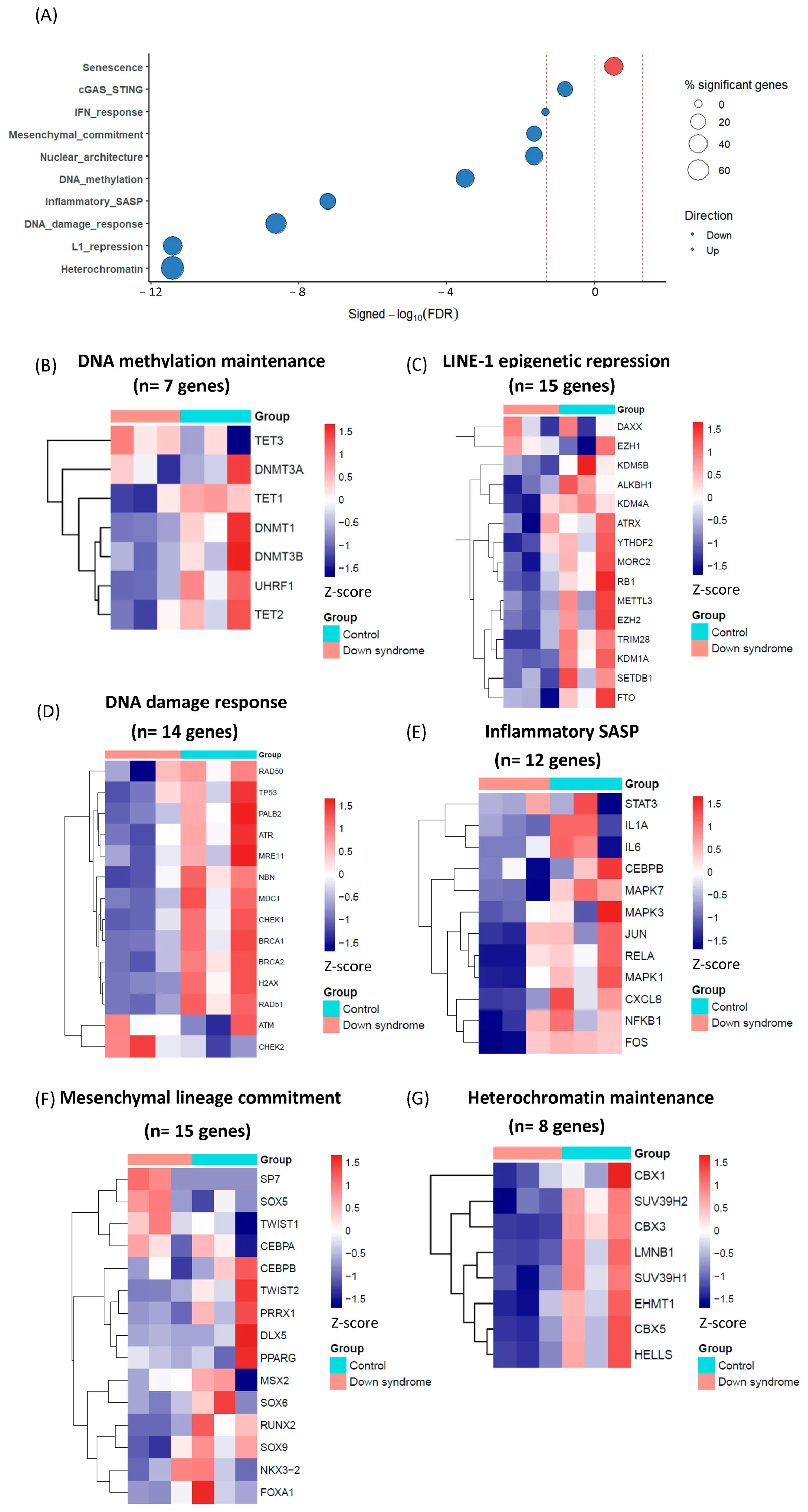
Functional pathway alterations and expression profiles of genes associated with transposable element dysregulation in Down syndrome. Bubble plot showing the direction and statistical significance of alterations in selected biological pathways in DS-MSCs compared with CTRLs. The x-axis represents the signed −log10(FDR), where positive and negative values indicate upregulation and downregulation, respectively. The bubble size represents the percentage of significant genes within each pathway. (**A**) Heatmaps showing the expression patterns of genes associated with (**B**) DNA methylation maintenance (*n* = 7 genes), (**C**) L1 epigenetic repression (*n* = 15 genes), (**D**) the DNA damage response (*n* = 13 genes), (**E**) the inflammatory senescence-associated secretory phenotype (SASP) (*n* = 12 genes), (**F**) mesenchymal lineage commitment (*n* = 15 genes), and (**G**) heterochromatin maintenance (*n* = 8 genes) in mesenchymal stem cells from individuals with DS and controls (CTRLs). Expression values are displayed as row-scaled normalized expression levels (z-scores), with red and blue indicating higher and lower relative expression, respectively. Hierarchical clustering of genes is illustrated by the dendrograms, while the annotation bar identifies samples according to their experimental group.

**Figure 6:**
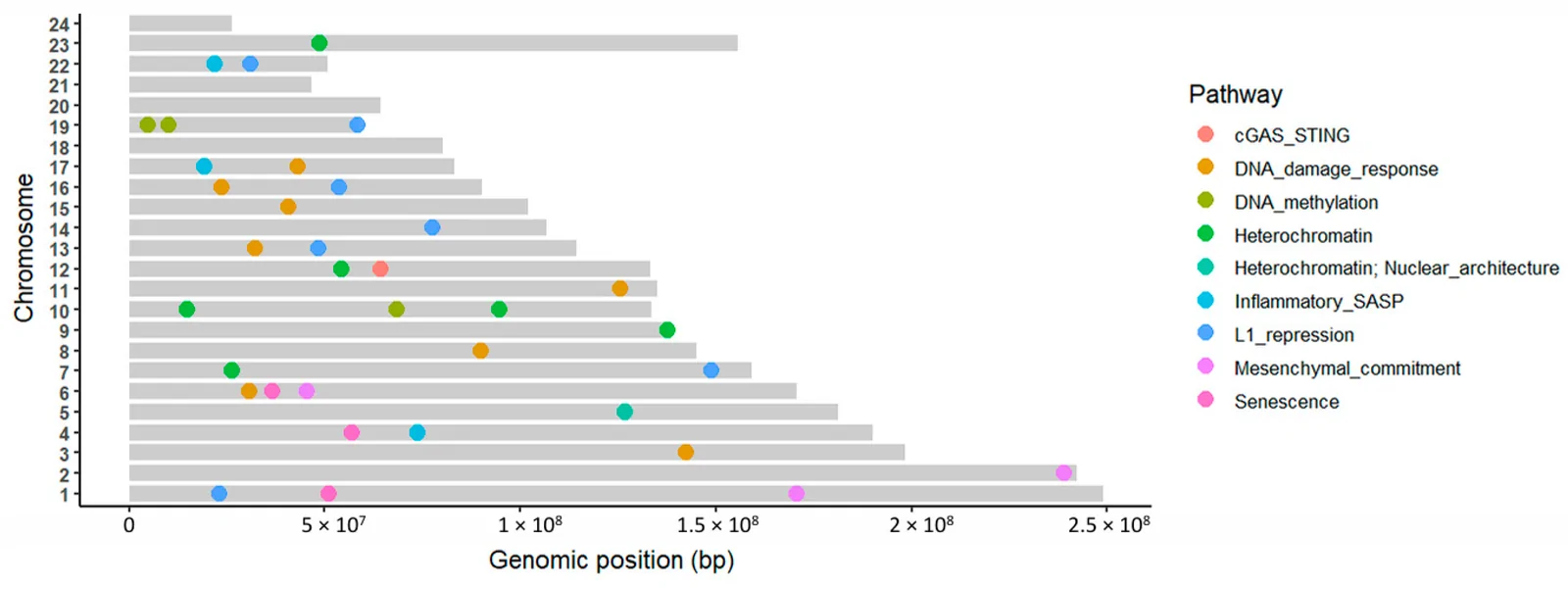
Chromosomal distribution of pathway-associated genes. Genomic mapping of genes associated with transcriptionally altered pathways. Gray horizontal bars represent individual chromosomes, with their length corresponding to chromosome size, while dots indicate the genomic position of pathway-associated genes. The dot colors correspond to the respective functional pathways.

## Data Availability

The data that support the findings of this study are available in the Gene Expression Omnibus (GEO), accession numbers GSE184450.

## Supplementary Materials

The following supporting information can be downloaded at https://www.mdpi.com/article/10.3390/ijms27219747/s1.

## Abbreviations

The following abbreviations are used in this manuscript:

CV-MSCs	Chorionic villi
CV	Chorionic villi
cntTable	Count matrix
CPM	Counts Per Million
DDR	DNA damage response
DS	Down syndrome
DS-MSCs	Down syndrome mesenchymal stem cells
FDR	False discovery rate
GEO	Gene Expression Omnibus
HSA21	Human chromosome 21
log2FC	log2 fold change
LINE-1 or L1	Long interspersed nuclear elements 1
MSCs	Mesenchymal stem cells
MSigDB	Molecular Signatures Database
REs	Repetitive elements
RIN	RNA integrity number
SASP	Senescence-Associated Secretory Phenotype
TEs	Transposable elements
T21	Trisomy 21
