# Supplementary material for: Transposable Element Dysregulation in Fetal Down Syndrome Mesenchymal Stem Cells: Insights from a Model of Premature Ageing
Source: Int J Mol Sci. 2026 Oct 30;27(21):9747. doi: 10.3390/ijms27219747 (PMC13762726; doi:10.3390/ijms27219747)
Supplement: Supplementary file 1 [file ijms-27-09747-s001.zip › ijms-4606249-supplementary.pdf]

International Journal of
Molecular Sciences

MDPI

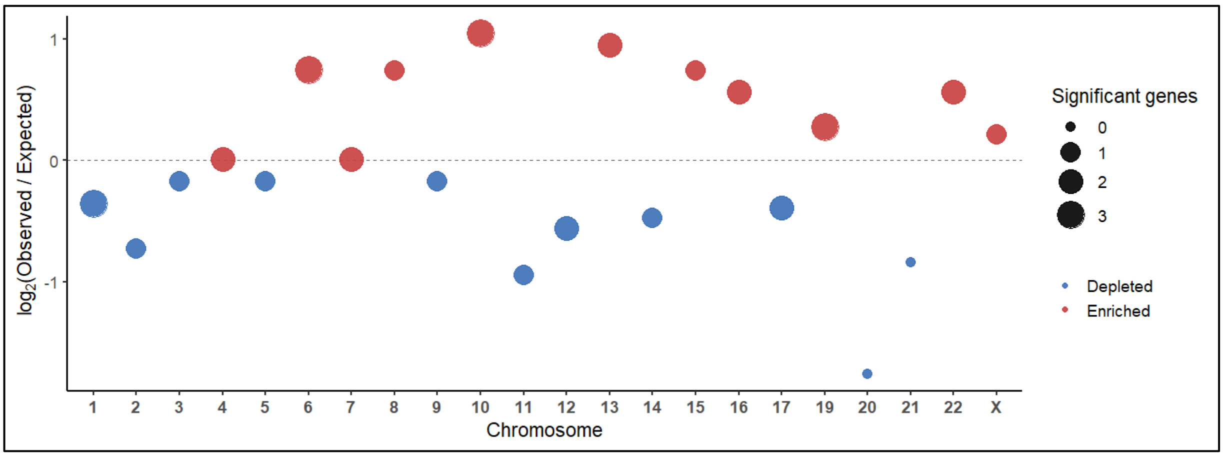

Figure S1. Chromosome-specific distribution of transcriptional enrichment and depletion. Chromosome-level analysis showing the balance between observed and expected transcriptional alterations across chromosome. The y-axis represents the $\log_2$ ratio of observed to expected events, with positive values indicating enrichment (red) and negative values indicating depletion (blue). The dashed horizontal line indicates the expected distribution ($\log_2$ observed/expected = 0). Point size is proportional to the number of significant genes associated with each chromosome.
